# Supplementary figures and images for: Transposon mutagenesis in Mycobacterium abscessus identifies an essential penicillin-binding protein involved in septal peptidoglycan synthesis and antibiotic sensitivity
Source: eLife. 2022 Jun 6;11:e71947. doi: 10.7554/eLife.71947 (PMC9170245; doi:10.7554/eLife.71947)

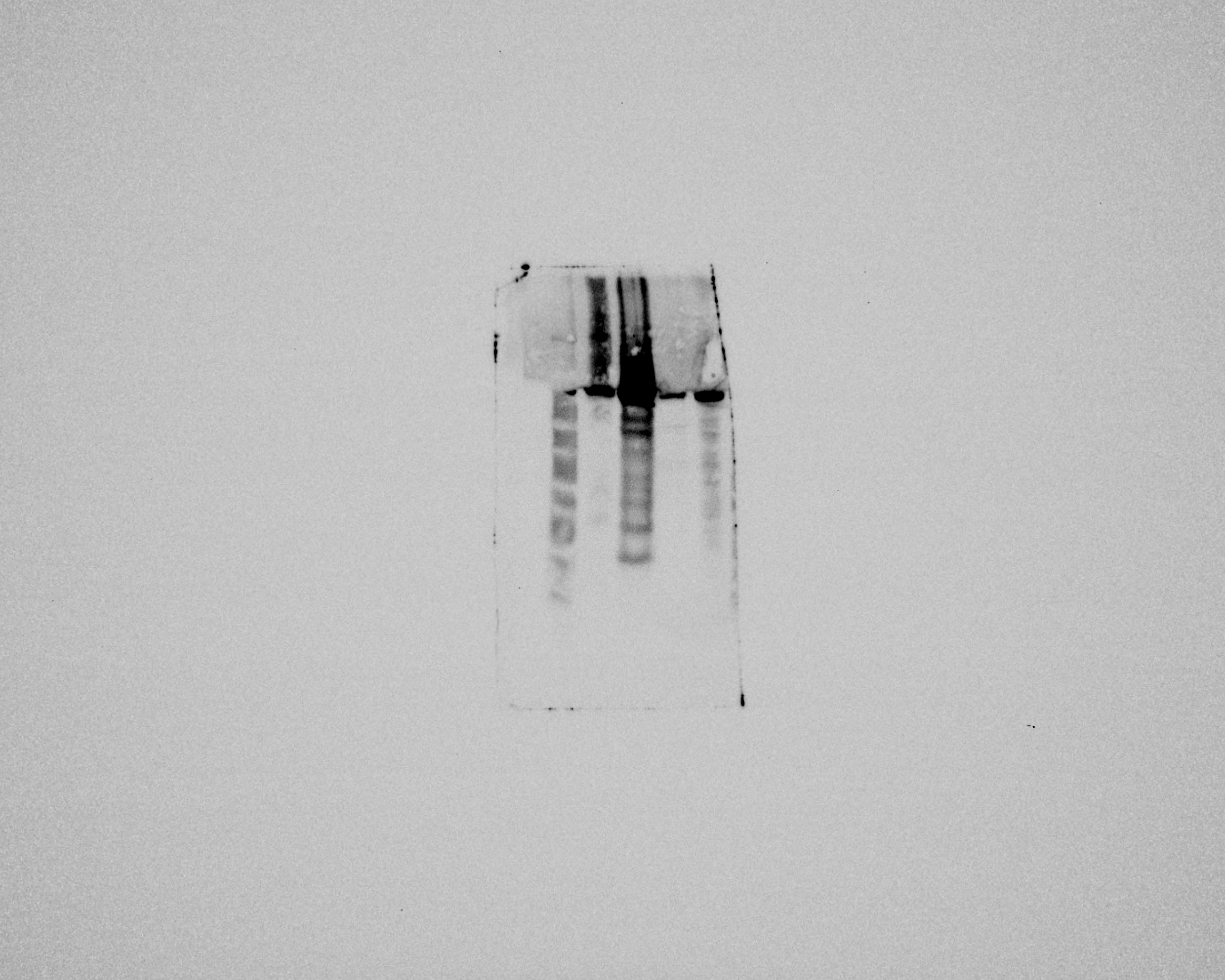

Supplement: Figure 3—figure supplement 3—source data 1. [file elife-71947-fig3-figsupp3-data1.zip › Figure3_supplementalFigure3/Figure3_figure supplement3B-source data 1.jpeg]

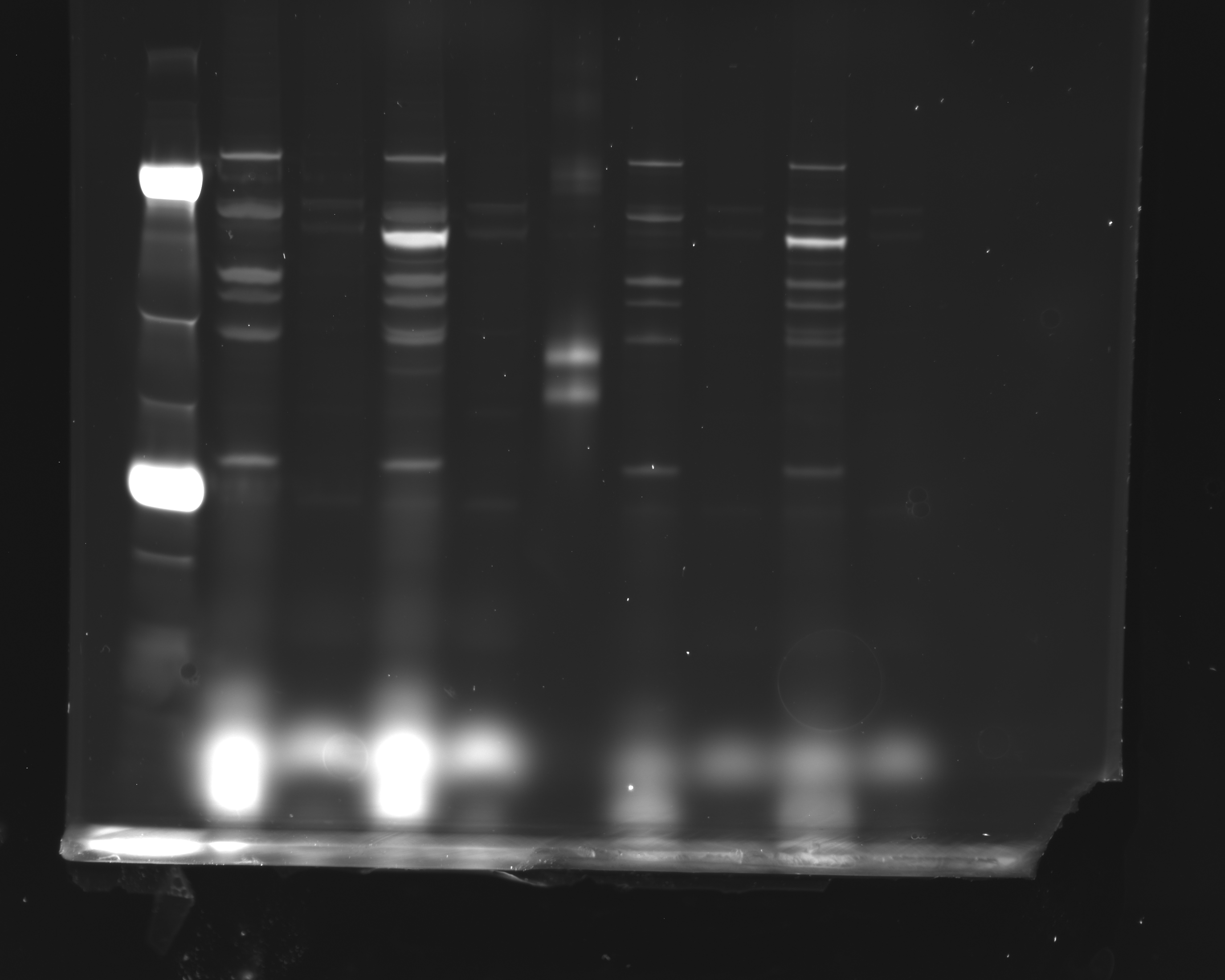

Supplement: Figure 3—figure supplement 3—source data 1. [file elife-71947-fig3-figsupp3-data1.zip › Figure3_supplementalFigure3/Figure3_figure supplement3A-source data 1.tiff]

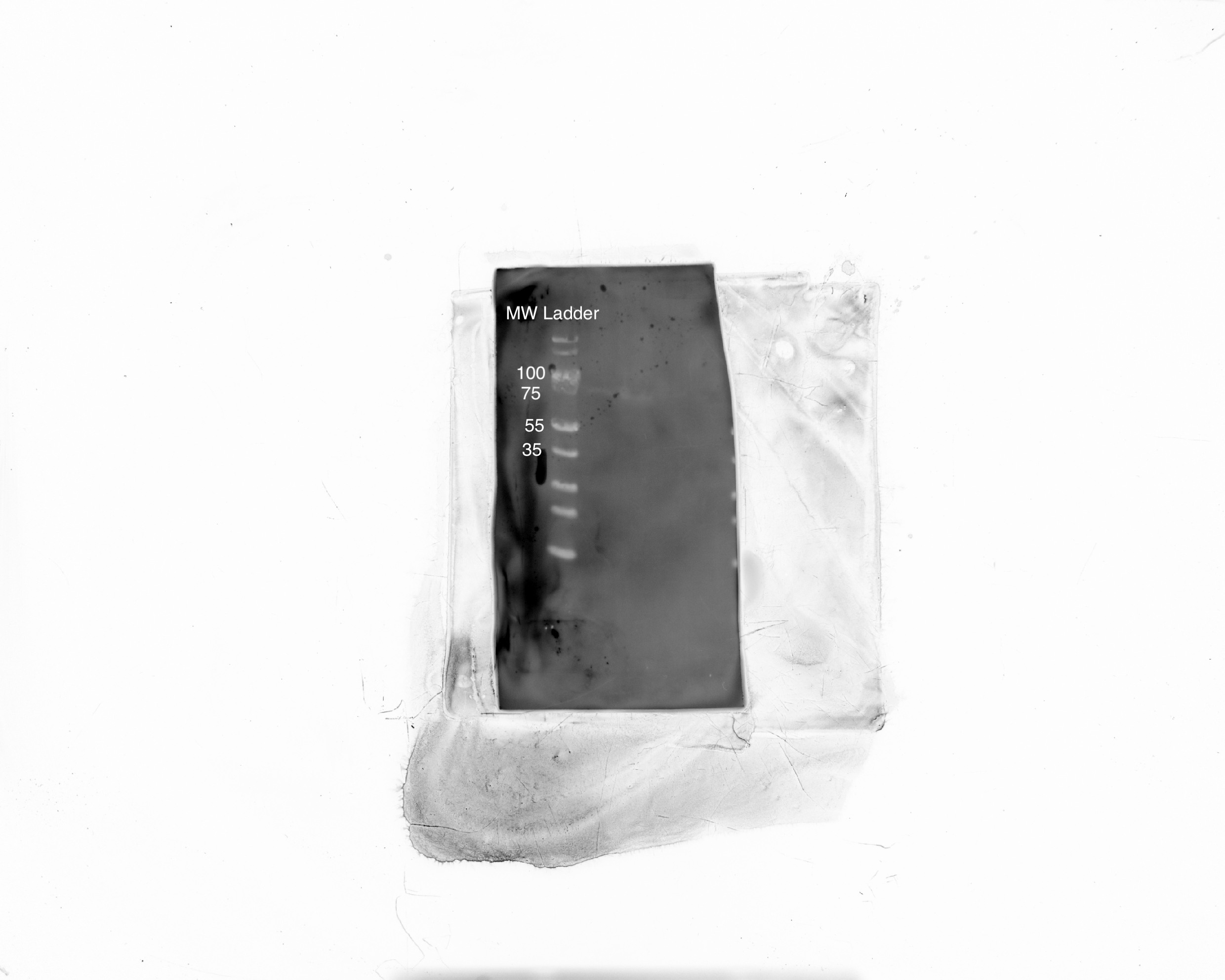

Supplement: Figure 3—figure supplement 3—source data 1. [file elife-71947-fig3-figsupp3-data1.zip › Figure3_supplementalFigure3/Figure3_figure supplement3B-source data 2_labeled.jpeg]

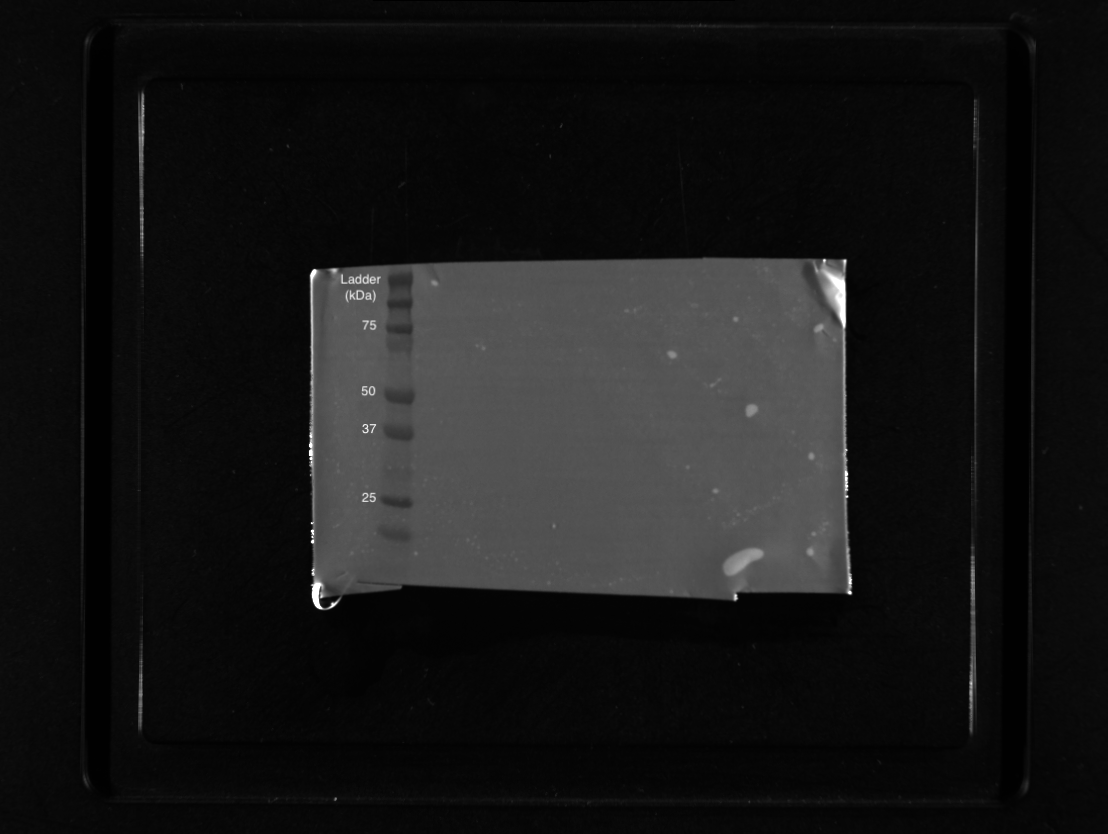

Supplement: Figure 3—figure supplement 3—source data 1. [file elife-71947-fig3-figsupp3-data1.zip › Figure3_supplementalFigure3/Figure3_figure supplement3A-source data 3_labeled.tif]

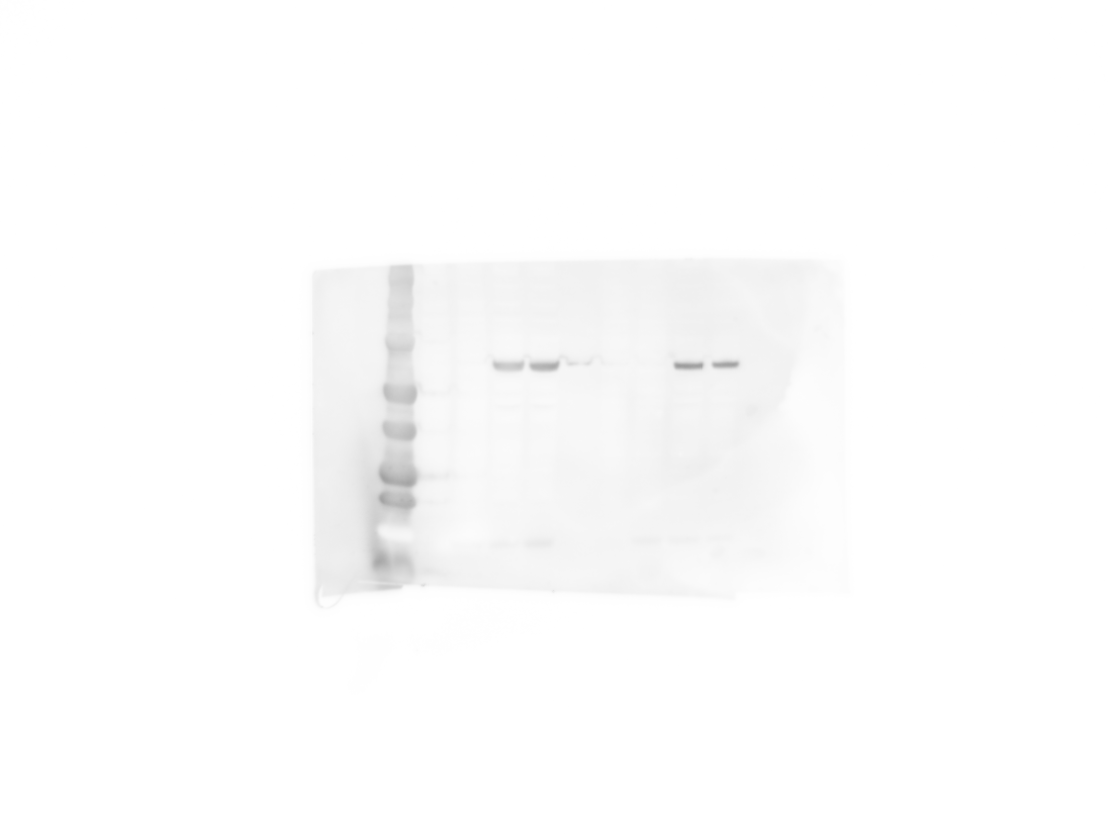

Supplement: Figure 3—figure supplement 3—source data 1. [file elife-71947-fig3-figsupp3-data1.zip › Figure3_supplementalFigure3/Figure3_figure supplement3A-source data 2.tif]

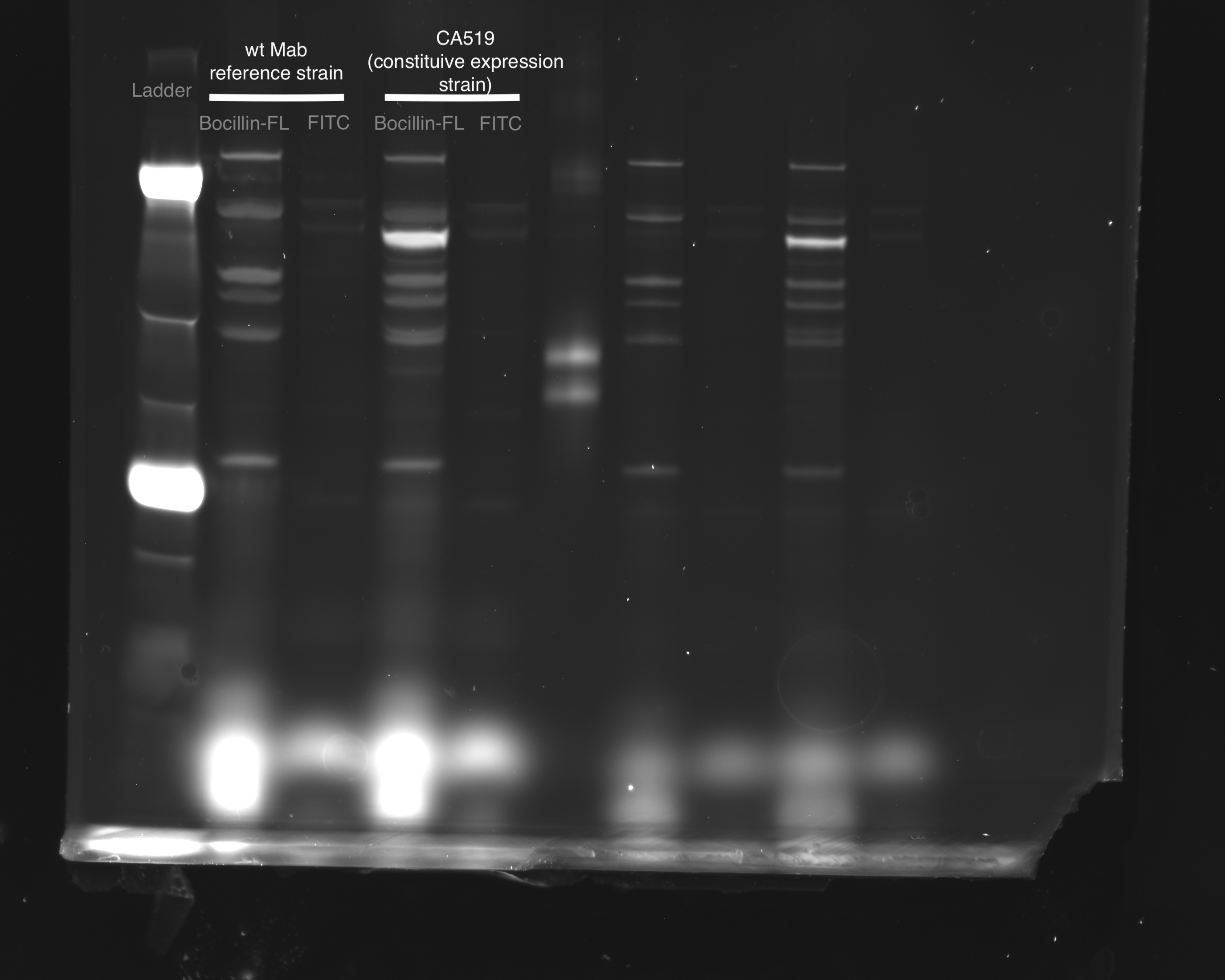

Supplement: Figure 3—figure supplement 3—source data 1. [file elife-71947-fig3-figsupp3-data1.zip › Figure3_supplementalFigure3/Figure3_figure supplement3A-source data 1_labeled.tiff]

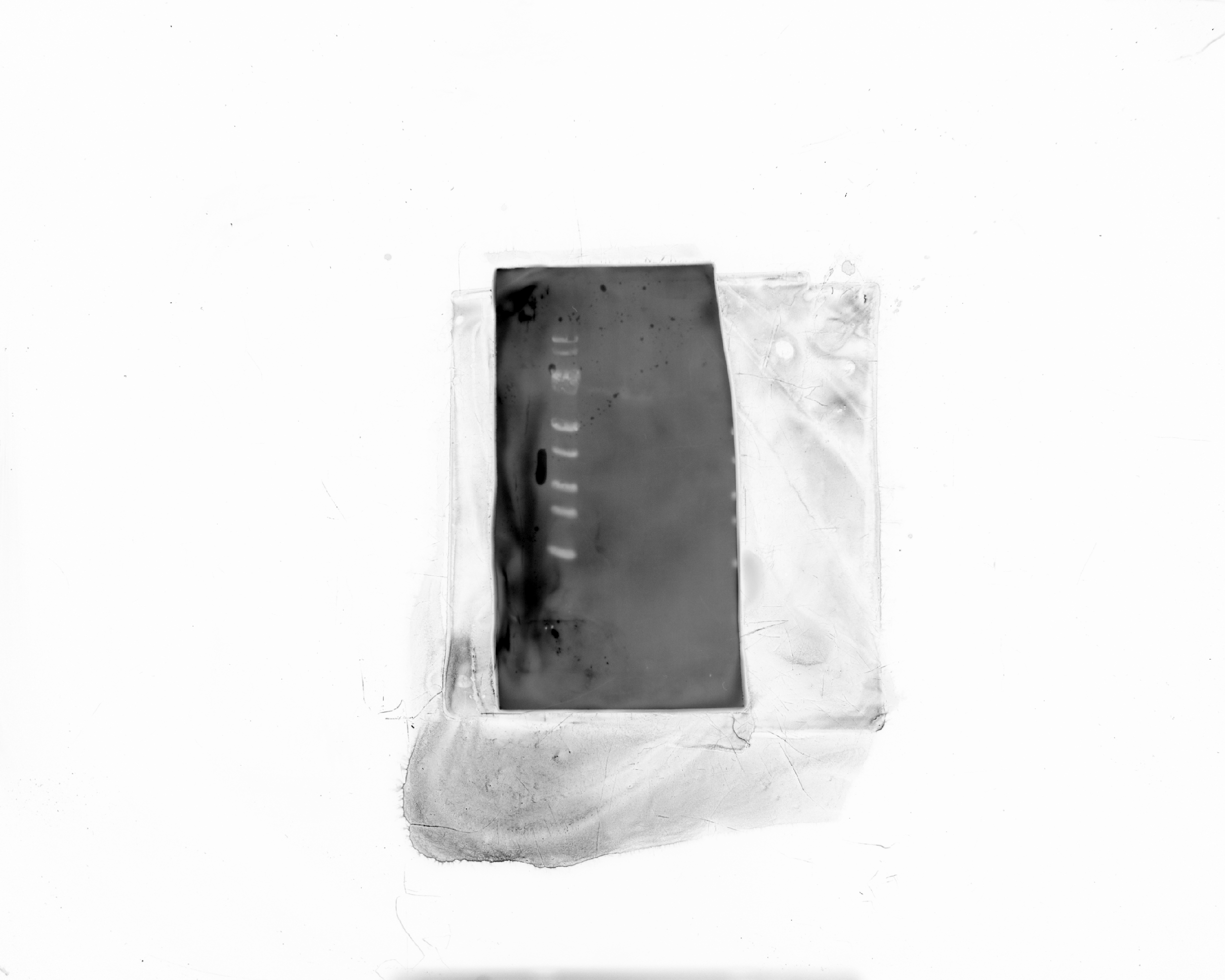

Supplement: Figure 3—figure supplement 3—source data 1. [file elife-71947-fig3-figsupp3-data1.zip › Figure3_supplementalFigure3/Figure3_figure supplement3B-source data 2.jpeg]

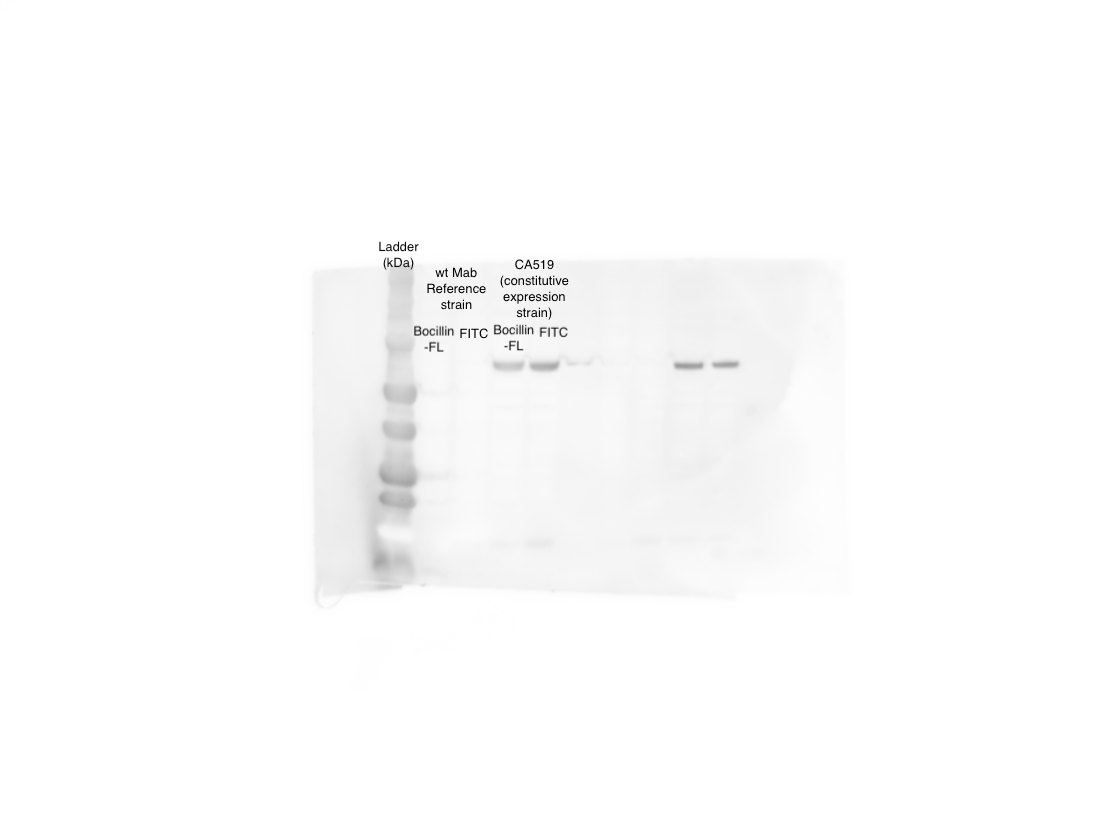

Supplement: Figure 3—figure supplement 3—source data 1. [file elife-71947-fig3-figsupp3-data1.zip › Figure3_supplementalFigure3/Figure3_figure supplement3A-source data 2_labeled.tif]

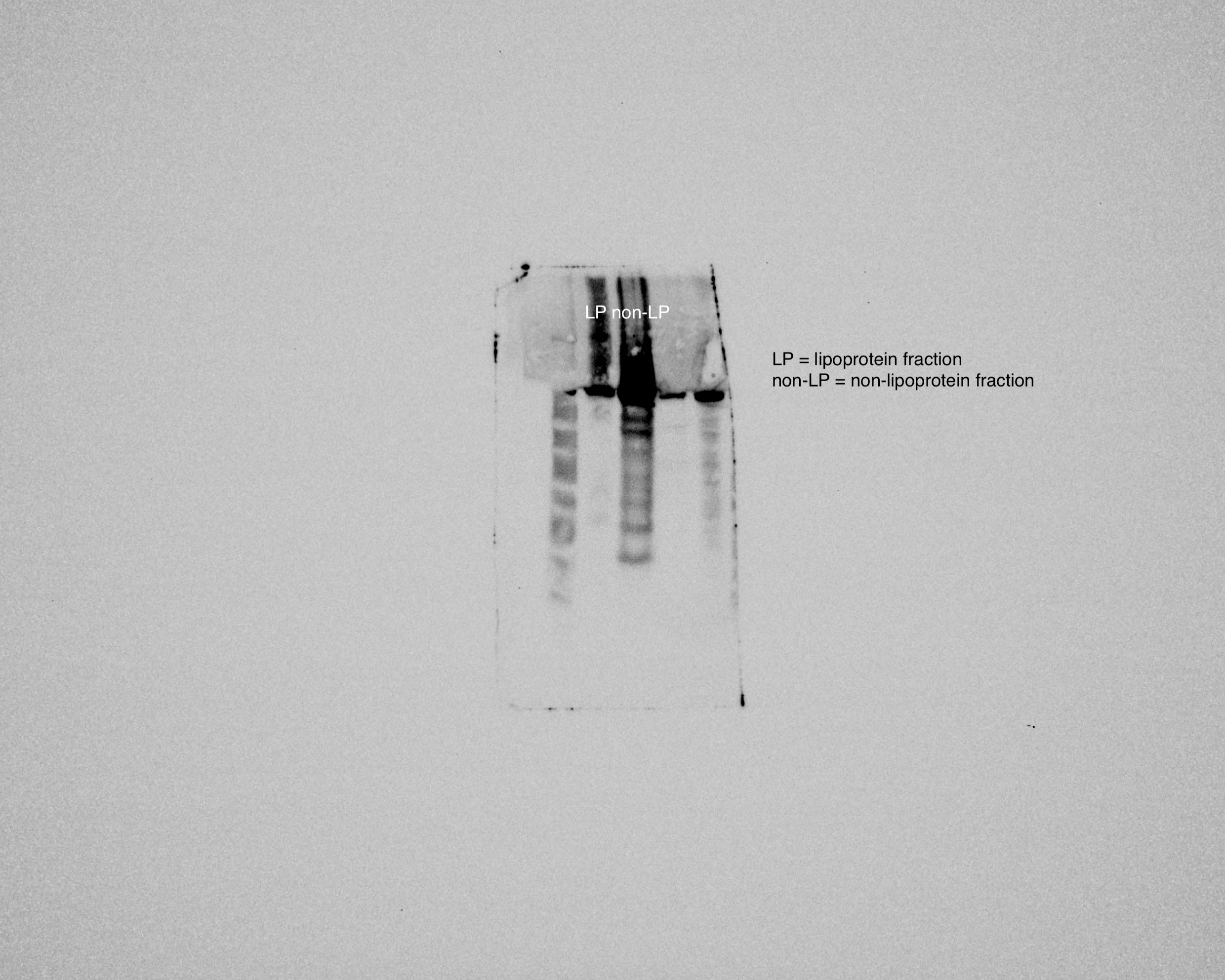

Supplement: Figure 3—figure supplement 3—source data 1. [file elife-71947-fig3-figsupp3-data1.zip › Figure3_supplementalFigure3/Figure3_figure supplement3B-source data 1_labeled.jpeg]

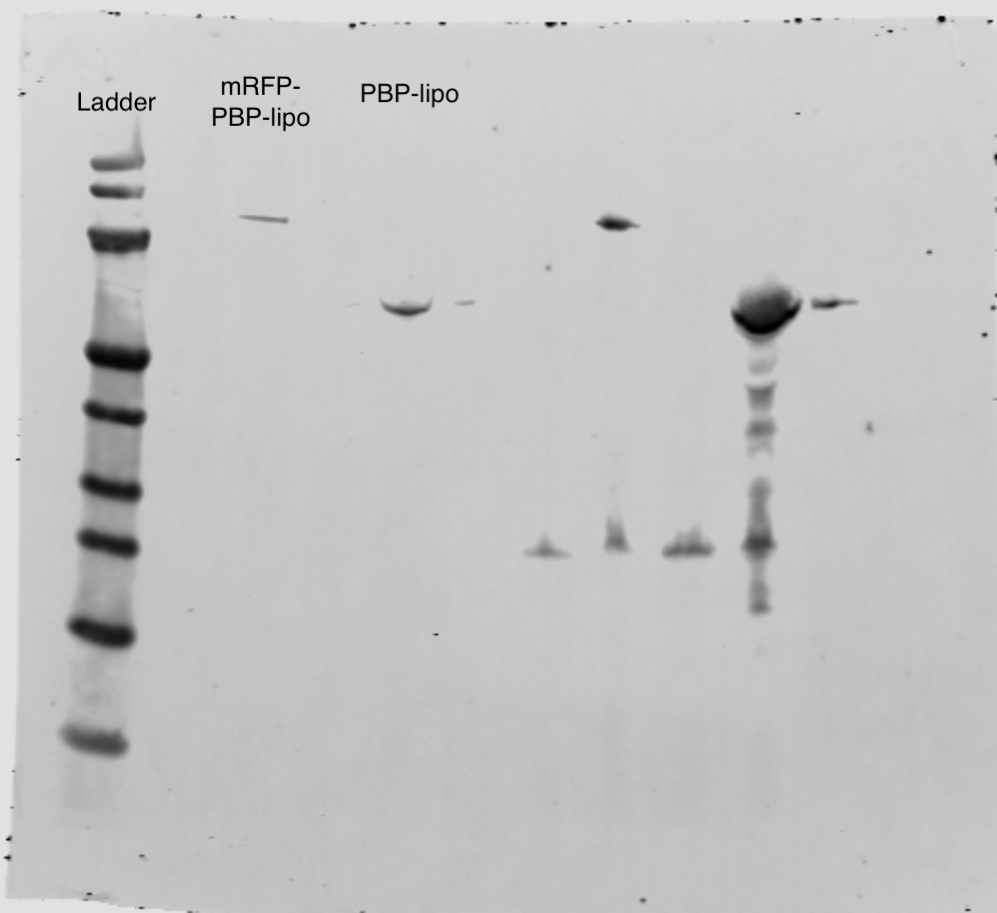

Supplement: Figure 5—figure supplement 1—source data 1. [file elife-71947-fig5-figsupp1-data1.zip › Figure5_figure_supplement1/Figure5_figure_supplement1_Source Data_labeled.png]

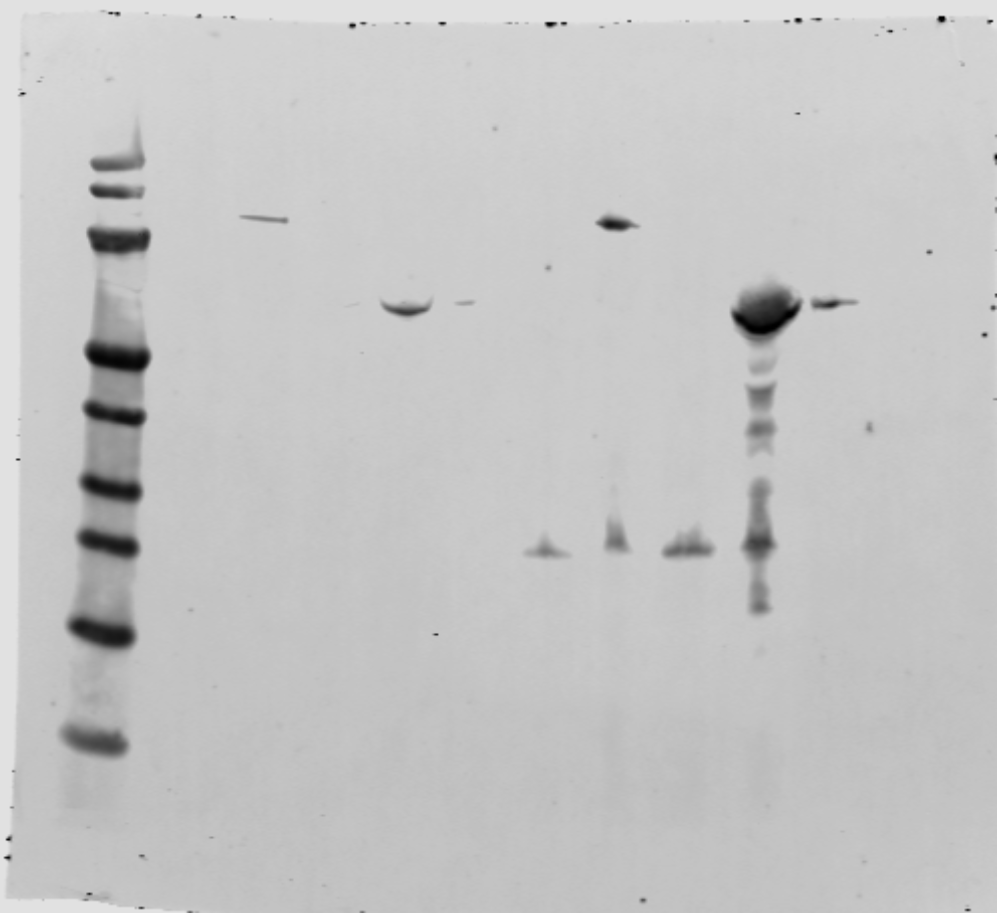

Supplement: Figure 5—figure supplement 1—source data 1. [file elife-71947-fig5-figsupp1-data1.zip › Figure5_figure_supplement1/Figure5_figure_supplement1_Source Data.png]

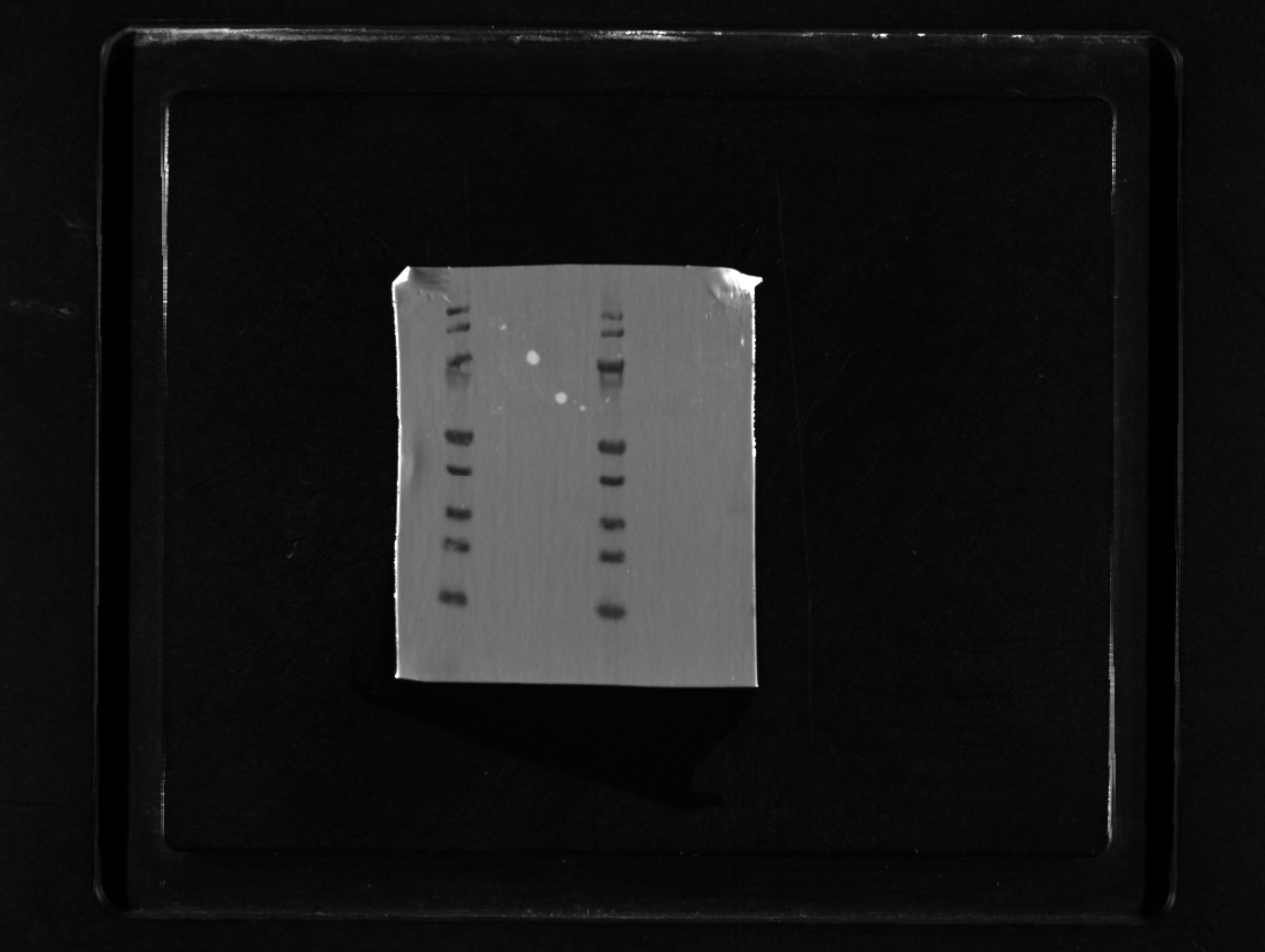

Supplement: Figure 6—figure supplement 4—source data 1. [file elife-71947-fig6-figsupp4-data1.zip › Figure6_figure_supplement4/Figure6_figure_supplement4_Source Data2_Labeled.tif]

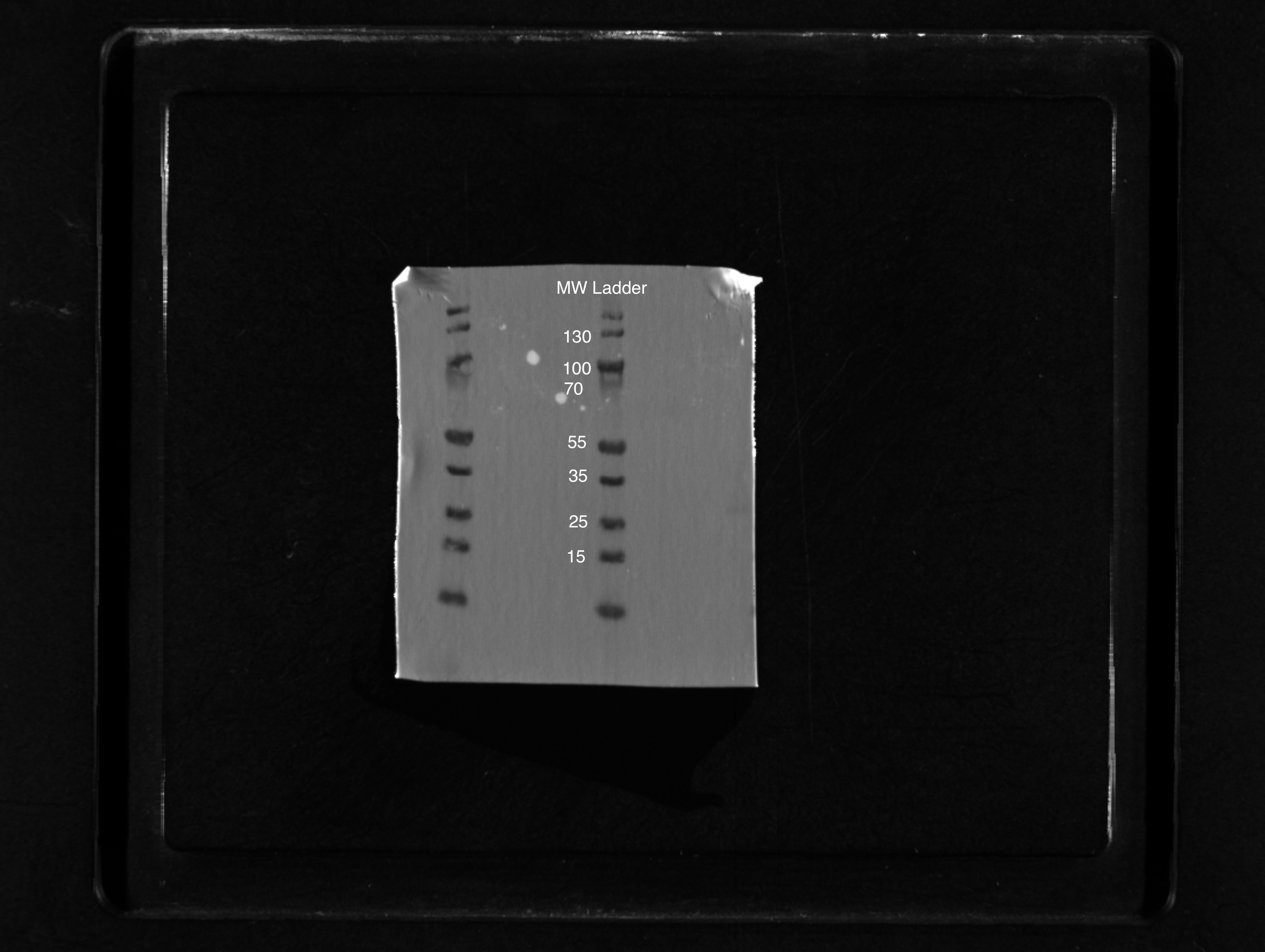

Supplement: Figure 6—figure supplement 4—source data 1. [file elife-71947-fig6-figsupp4-data1.zip › Figure6_figure_supplement4/Figure6_figure_supplement4_Source Data2.tif]

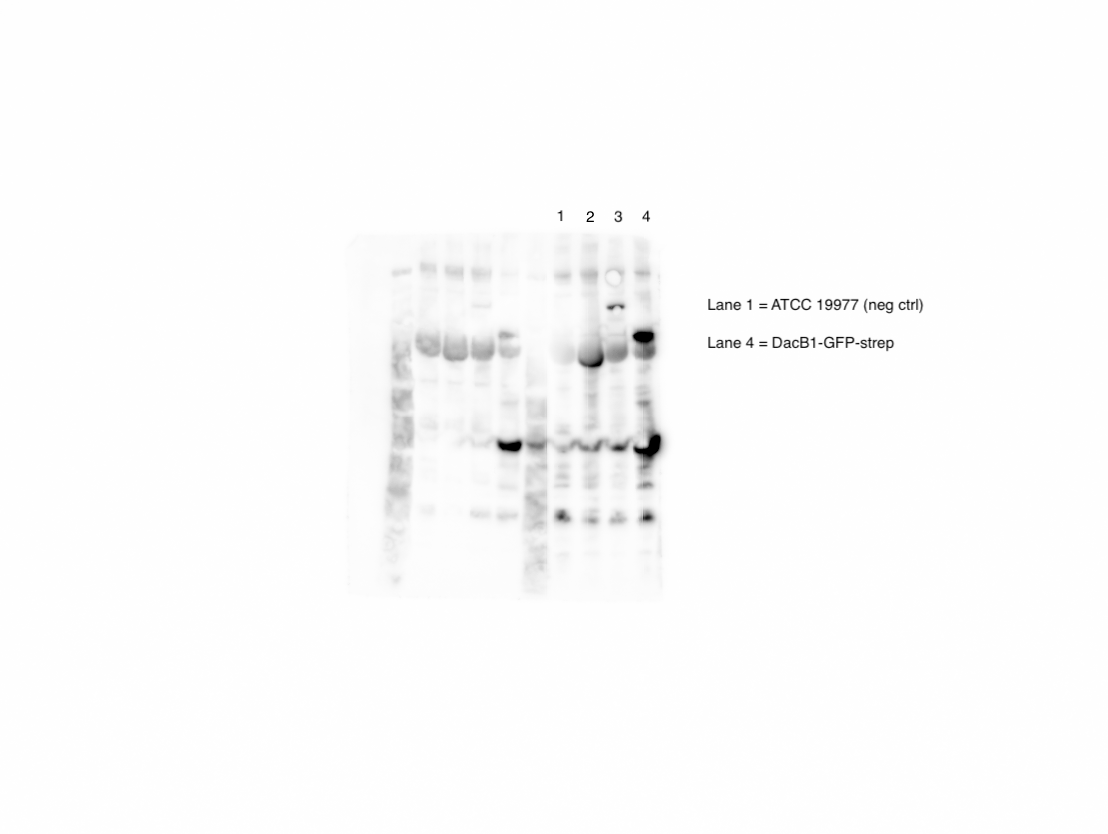

Supplement: Figure 6—figure supplement 4—source data 1. [file elife-71947-fig6-figsupp4-data1.zip › Figure6_figure_supplement4/Figure6_figure_supplement4_Source Data1_Labeled.tif]

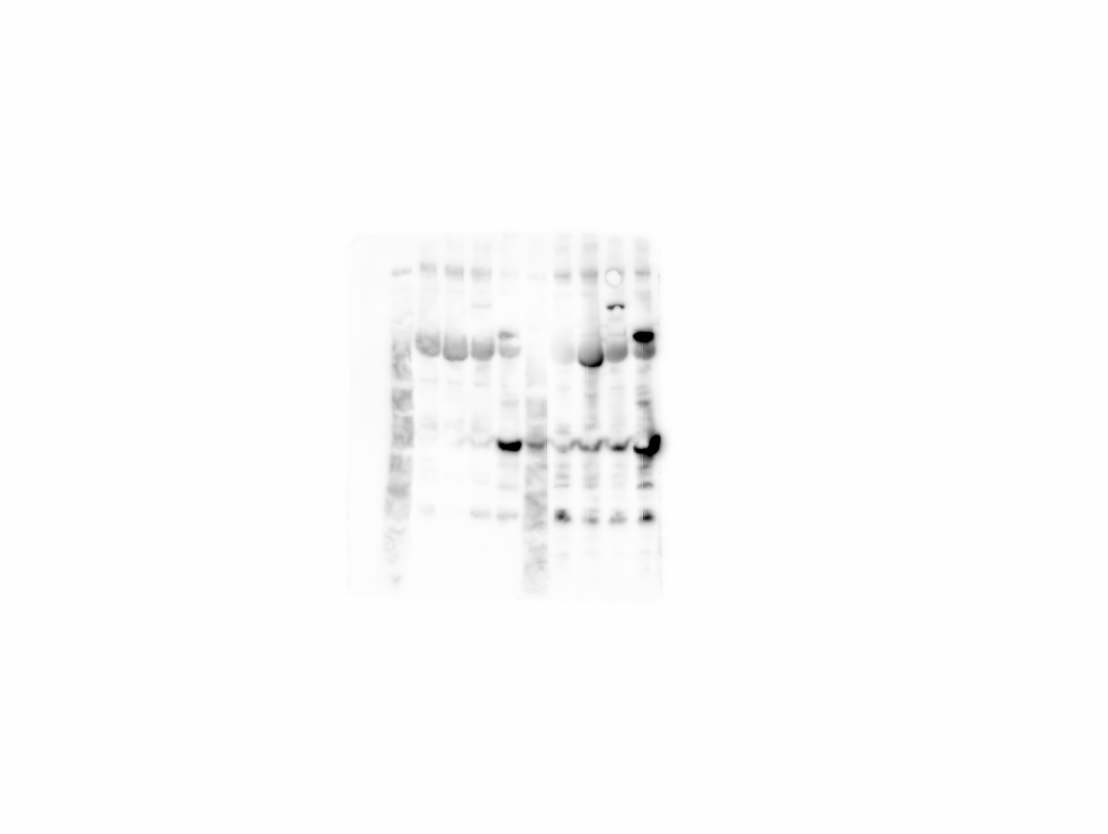

Supplement: Figure 6—figure supplement 4—source data 1. [file elife-71947-fig6-figsupp4-data1.zip › Figure6_figure_supplement4/Figure6_figure_supplement4_Source Data1.tif]
