# Supplementary material for: Transposon mutagenesis in Mycobacterium abscessus identifies an essential penicillin-binding protein involved in septal peptidoglycan synthesis and antibiotic sensitivity
Source: eLife. 2022 Jun 6;11:e71947. doi: 10.7554/eLife.71947 (PMC9170245; doi:10.7554/eLife.71947)
Supplement: Supplementary file 1. [file elife-71947-supp1.docx]

| **Supplementary Table 1: Summary of *Mab subsp. abscessus* ATCC 19977 TnSeq Libraries** | | |
| --- | --- | --- |
|  | **Total Counts** | **% Saturation** |
| Library #1 | 1176998 | 54.2 |
| Library #2 | 3600892 | 62.3 |
| Library #3 | 4080756 | 64.1 |
