# Supplementary material for: Transposon mutagenesis in Mycobacterium abscessus identifies an essential penicillin-binding protein involved in septal peptidoglycan synthesis and antibiotic sensitivity
Source: eLife. 2022 Jun 6;11:e71947. doi: 10.7554/eLife.71947 (PMC9170245; doi:10.7554/eLife.71947)
Supplement: Supplementary file 3. [file elife-71947-supp3.docx]

| **Supplementary Table 3: List of essential genes in *Mab* with *non-essential* orthologs in *Mtb*** | | | | | |
| --- | --- | --- | --- | --- | --- |
| ***Mab* ortholog** | **H37Rv ortholog** | **gene** | **E-value** | **% aa identity** | **annotation of H37Rv gene** |
| MAB_2129 | Rv2124c | metH | 1.00E-174 | 34 | 5-methyltetrahydrofolate--homocystein methyltransferase MetH (methionine synthase, vitamin-B12 dependent isozyme) (ms) |
| MAB_3675 | Rv3318 | sdhA | 0 | 90 | Probable succinate dehydrogenase (flavoprotein subunit) SdhA (succinic dehydrogenase) (fumarate reductase) (fumarate dehydrogenase) (fumaric hydrogenase) |
| MAB_3686c | Rv0066c | icd2 | 0 | 81 | Probable isocitrate dehydrogenase [NADP] Icd2 (oxalosuccinate decarboxylase) (IDH) (NADP+-specific ICDH) (IDP) |
| MAB_3167c | Rv2864c | - | 0 | 64 | Possible penicillin-binding lipoprotein |
| MAB_1920 | Rv2222c | glnA2 | 0 | 86 | Probable glutamine synthetase GlnA2 (glutamine synthase) (GS-II) |
| MAB_1662c | Rv2391 | sirA | 0 | 78 | Ferredoxin-dependent sulfite reductase SirA |
| MAB_1563 | Rv2474c | - | 9.00E-76 | 55 | hypothetical protein |
| MAB_1089 | Rv0996 | - | 5.00E-68 | 49 | Probable conserved transmembrane protein |
| MAB_4049c | Rv0489 | gpm1 | 1.00E-137 | 79 | Probable phosphoglycerate mutase 1 Gpm1 (phosphoglyceromutase) (PGAM) (BPG-dependent PGAM) |
| MAB_1484 | Rv1340 | rphA | 7.00E-149 | 85 | Probable ribonuclease RphA (RNase PH) (tRNA nucleotidyltransferase) |
| MAB_3602c | Rv3256c | - | 2.00E-99 | 51 | hypothetical protein |
| MAB_3110 | Rv2788 | sirR | 4.00E-125 | 78 | Probable transcriptional repressor SirR |
| MAB_3673 | Rv3316 | sdhC | 2.00E-58 | 75 | Probable succinate dehydrogenase (cytochrome B-556 subunit) SdhC (succinic dehydrogenase) (fumarate reductase) (fumarate dehydrogenase) (fumaric hydrogenase) |
| MAB_3995 | Rv0505c | serB1 | 8.00E-142 | 82 | Possible phosphoserine phosphatase SerB1 (PSP) (O-phosphoserine phosphohydrolase) (pspase) |
| MAB_2848c | Rv2552c | aroE | 4.00E-94 | 60 | Probable shikimate 5-dehydrogenase AroE (5-dehydroshikimate reductase) |
| MAB_1513 | Rv2523c | acpS | 5.00E-68 | 74 | holo-[acyl-carrier protein] synthase AcpS (holo-ACP synthase) (CoA:APO-[ACP]pantetheinephosphotransferase) (CoA:APO-[acyl-carrier protein]pantetheinephosphotransferase) |
| MAB_3676 | Rv3319 | sdhB | 8.00E-167 | 84 | Probable succinate dehydrogenase (iron-sulphur protein subunit) SdhB (succinic dehydrogenase) (fumarate reductase) (fumarate dehydrogenase) (fumaric hydrogenase) |
| MAB_4954c | Rv3923c | rnpA | 3.00E-27 | 51 | Ribonuclease P protein component RnpA (RNaseP protein) (RNase P protein) (protein C5) |
| MAB_1446 | Rv1303 | - | 3.00E-41 | 57 | hypothetical protein |
| MAB_4471 | Rv0236A | - | 3.00E-23 | 68 | Small secreted protein |
