# Supplementary material for: Transposon mutagenesis in Mycobacterium abscessus identifies an essential penicillin-binding protein involved in septal peptidoglycan synthesis and antibiotic sensitivity
Source: eLife. 2022 Jun 6;11:e71947. doi: 10.7554/eLife.71947 (PMC9170245; doi:10.7554/eLife.71947)
Supplement: Supplementary file 4. [file elife-71947-supp4.docx]

| **Supplementary Table 4: TnSeq Summary of *Msm* Libraries** | | | |
| --- | --- | --- | --- |
| **Strain** | **Total counts** | **Saturation** | **# of conditional essentials compared to wt** |
| mc^2^155 -1 | 1805936 | 0.519 | - |
| mc^2^155 -2 | 1264512 | 0.534 | - |
| ΔPBP-lipo-1 | 1389005 | 0.628 | 0 |
