# Supplementary material for: Transposon mutagenesis in Mycobacterium abscessus identifies an essential penicillin-binding protein involved in septal peptidoglycan synthesis and antibiotic sensitivity
Source: eLife. 2022 Jun 6;11:e71947. doi: 10.7554/eLife.71947 (PMC9170245; doi:10.7554/eLife.71947)
Supplement: Supplementary file 6. [file elife-71947-supp6.docx]

| **Supplementary Table 6: MIC (µg/ml) of wildtype H37Rv &** $\boldsymbol{\Delta}$**PBP-lipo** | | | |
| --- | --- | --- | --- |
|  | MIC | |  |
| **Antibiotic** | **wt H37Rv** | $\boldsymbol{\Delta}$**PBP-lipo** | **Fold Difference** |
| **Cell Wall** |  |  |  |
| Ampicillin | 8 | 16 | 0.5 |
| Amoxicillin | 4 | 4 | 1 |
| Faropenem | >64 | >64 | N/A |
| Cefoxitin | 32 | 32 | 1 |
|  |  |  |  |
| **Ribosome** |  |  |  |
| Clarithromycin | 0.25 | <0.125 | N/A |
| Erythromycin | 4 | 1 | 4 |
| Amikacin | <0.125 | 0.125 | N/A |
|  |  |  |  |
| **RNA Polymerase** |  |  |  |
| Rifampicin | 16 | 8 | 2 |
|  |  |  |  |
| **DNA Gyrase** |  |  |  |
| Ofloxacin | 0.25 | 0.25 | 1 |
